# The Role of Smallholder Farming on Rural Household Dietary Diversity

**DOI:** 10.3390/agriculture13030595

**Published:** 2023-02-28

**Authors:** Simphiwe Innocentia Hlatshwayo, Rob Slotow, Mjabuliseni Simon Cloapas Ngidi

**Affiliations:** 1 African Centre for Food Security, School of Agricultural, Earth and Environmental Sciences, College of Agriculture, Engineering and Science, University of KwaZulu-Natal, Private Bag X01, Scottsville, Pietermaritzburg 3201, South Africa; 2 Centre for Transformative Agricultural and Food Systems, School of Agricultural, Earth and Environmental Sciences, College of Agriculture, Engineering and Science, University of KwaZulu-Natal, Private Bag X01, Scottsville, Pietermaritzburg 3201, South Africa; 3 Centre for Transformative Agricultural and Food Systems, School of Life Sciences, College of Agriculture, Engineering and Science, University of KwaZulu-Natal, Private Bag X01, Scottsville, Pietermaritzburg 3201, South Africa; 4 Department of Agricultural Extension and Rural Resource Management, School of Agricultural, Earth and Environmental Sciences, College of Agriculture, Engineering and Science, University of KwaZulu-Natal, Private Bag X01, Scottsville, Pietermaritzburg 3201, South Africa

**Keywords:** subsistence farming, crop productivity, market participation, smallholder farmers, dietary diversity

## Abstract

The importance of smallholder farming is increasingly recognized in rural areas where increased crop productivity and market participation can effectively improve their dietary diversity and nutrition quality. However, rural households are still faced with severe food insecurity and malnutrition. The study sought to assess the role of smallholder farming in crop productivity and market access on rural household dietary diversity. The secondary data were collected using a quantitative research method, and 1520 participants were selected using a stratified random sampling technique. The descriptive results showed that cereals were the most (98%) consumed food group, while vegetables and fruits were the least consumed food groups, at 37% and 23%, respectively. The results from the Household Dietary Diversity Score (HDDS) showed that 57% of smallholder farmers consumed highly diverse diets (more or equal to six food groups), whereas 25% and 18% of smallholder farmers consumed medium dietary diversity (four to five food groups) and low diverse diets (less or equal to three food groups), respectively. The findings from the Conditional Mixed Process (CMP) and Poisson endogenous treatment effect models showed that household size, ownership of livestock, wealth index, and involvement in crop production positively influenced household dietary diversity. On the other hand, output and access to market information showed a negative effect. Social grants had contradicting effects: they had a negative impact on the HDDS received from crop productivity while they had a positive effect on the HDDS from market participation. Providing different ways smallholder farmers can use their funds effectively can help improve household dietary diversity and nutrition quality. The study recommended that more workshops and training be conducted that cover all the sustainable production systems that smallholder farmers can undertake to produce different food groups. These will raise awareness among smallholder farmers about the requirements for balanced diets for food and nutrition security.

## 1 Introduction

South Africa is one of the developing countries that is faced with significant food and nutrition challenges. The country suffers from malnutrition, obesity, persistent hunger, and chronic diseases [1,2]. Undernutrition is relatively high among young children, while obesity is relatively high among adults and older children. Approximately 68% of South African women and 31% of South African men were reported obese or overweight in 2022 [3]. In 2021, almost 27% of children under the age of five were stunted [4]. Most people affected by undernutrition and malnutrition live in rural areas of South Africa [5,6]. Extreme conditions in rural areas, such as a rapid increase in population rate, extreme poverty, high unemployment, and inequality, worsen the health issues affecting rural households [6]. These conditions have an enormous impact on adequate access to quality food and nutrients, limiting rural areas to few food choices, mainly consisting of starchy staples such as maize and potatoes [7]. Muthini et al. [8] stated that dietary diversity is essential for a balanced and healthy diet beyond food quantity.

Dietary diversity has become a familiar food and nutrition security indicator, which reflects the different kinds of foods consumed by individuals and households [9]. At the household level, dietary diversity scores are considered a food security indicator. In contrast, at an individual level, the dietary diversity scores are substitutes for dietary quality and nutrition as they are linked with nutrition status and micronutrient intake [9,10]. Existing literature indicates that increasing crop productivity and market participation could effectively improve dietary diversity and nutrition quality among smallholder farmers [8,11,12]. Muthini and Nzuma [8] mentioned two main pathways by which smallholder farmers acquire their food, which are (i) own production (the smallholder pathway) and (ii) market purchases (the market pathway). The subsistence pathway recommends that smallholder farmers increase their crop productivity and production diversity to ensure the direct availability of different food items for home consumption [11]. As smallholder house-holds typically consume large proportions of what they produce at home, higher farm crop productivity may also lead to higher dietary diversity. The market pathway recommends that smallholder farmers produce more diverse crops to enable them to participate in the market and achieve stable incomes that households can use to buy diverse food items [13].

According to the Sustainable Development Goals (SDGs), agriculture has a potential role in improving nutrition; SDG 2 emphasizes the need to improve productivity among smallholder farmers [14,15]. The main objective is to improve the quantity and quality of smallholder households’ diets. However, the question is how exactly this can be achieved. Some studies [16–18] suggested a need to focus mainly on increasing production among smallholder farmers, whereas others emphasize increasing smallholders’ access to the market [19–22]. Therefore, smallholder farming is a vital entry point for policy interventions to improve food and nutrition security. However, while agricultural interventions have reduced poverty and created job opportunities and economic growth, evidence for nutrition improvement is minimal [23–25]. The importance of subsistence farming for increasing household dietary diversity through crop productivity and market access pathways has yet to be rigorously investigated in South Africa. Sinyolo et al. [2] investigated the role of diversified farm production in dietary diversity among subsistence agricultural communities in South Africa; however, that study did not look at market access. Muthini et al. [8] investigated the subsistence and market pathways of household dietary diversity in Kenya. Koppmair et al. [21] analyzed the impact of farm production and market access on dietary diversity in Malawi. Therefore, this study aims to address the research gap by using the data from smallholder households in South Africa to assess the importance of smallholder farming, in terms of both crop productivity and market access pathways, for rural household dietary diversity.

## 2 Material and Methods

### 2.1 Description of Study Areas

The study relied on secondary data gathered from two South African provinces (Mpumalanga and Limpopo). These provinces were selected in this study because they are listed as unprivileged provinces with high populations. The provinces are also characterized by a lack of job opportunities. While the smallholder farmers predominantly live in lands under the ownership of tribal authorities, they use their resources to produce for their own survival and for sale to improve their livelihoods. In these provinces, starchy staple crops such as maize, beans, and potatoes are mainly produced, meaning that crop diversification is minimal [26,27]. This makes households more vulnerable to malnutrition and food insecurity.

Mpumalanga province is divided into two regions: the subtropical Lowveld plains and the Highveld escarpment. The Highveld is substantially cooler due to its elevation of 1700–2300 m above sea level, but the Lowveld is subtropical. The Lowveld is hot in the summer and warm in the winter, whereas the Highveld is hot in the summer but cold in the winter [28]. The daily temperatures in the province range between 6 and 20 degree Celsius in the winter and between 20 and 38 degrees Celsius in the summer [29]. Sugar production, crops such as wheat, maize, potatoes, sunflowers, almonds, subtropical and tropical fruit, and cattle production are all part of the province [27], where smallholder farmers mainly produce maize, potatoes, and cattle.

Limpopo province has a subtropical climate, with most rain falling during the summer (October to March). Annual rainfall in the province fluctuates between 300, 400, and 600 mm. The average temperature ranges from 0 °C to 20 °C in the winter and from 7 °C to 27 °C in the summer [30]. Mornings are quite chilly and dry during the winter, with sunny days, while nights are normally frost-free and frigid [31]. The province is one of the country’s most important farming areas, producing cereals, fruits, vegetables, and tea [32].

### 2.2 Data Collection Method

The secondary data utilized in this study were obtained during the 2016/2017 growing season. A questionnaire with both closed and open-ended questions was used to interview smallholder farmers. The multi-stage stratified random sampling methodology was utilized to select a total of 1520 smallholder farmers for the quantitative research method. According to Welman et al. [33], in stratified random sampling, the population is formed of multiple distinguishable, non-overlapping sub-populations (named strata or singularly strata) that differ from one another in terms of specified variables. The benefit of stratified random sampling is that it ensures all relevant strata are sufficiently represented, but it is less expensive and takes less time [33]. Mpumalanga was separated into four districts for this study, and Limpopo was divided into three districts. The districts were separated using socioeconomic factors, such as gender and age. The data were compiled by the National Department of Agriculture, Land Reform, and Rural Development (DALRRD) under the auspices of the South African Vulnerability Assessment Committee (SAVAC).

### 2.3 Conceptual Framework

**Figure 1:**
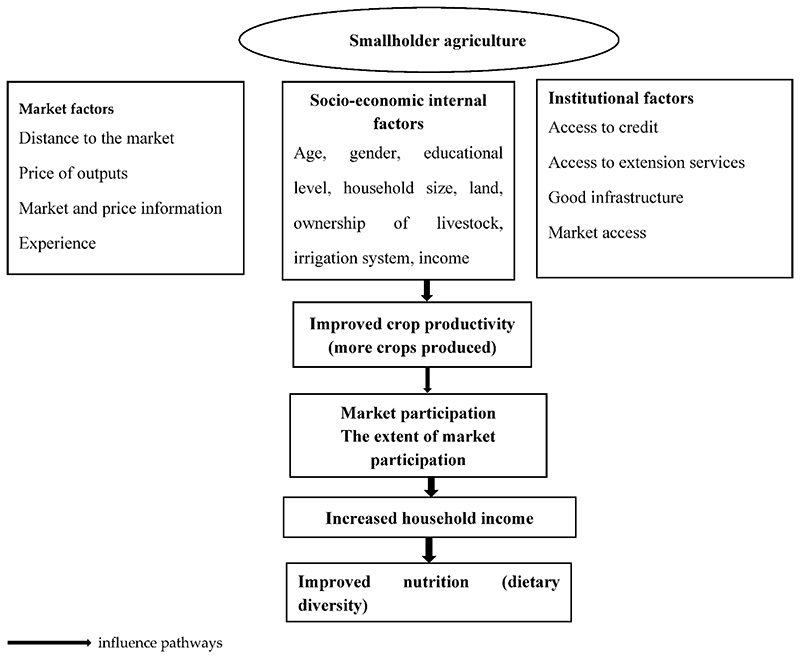
Diagrammatic representation of the conceptual framework.

The main goal of market access and improved crop productivity is to enhance house-hold dietary diversity, resulting in sustainable household livelihoods. It is, therefore, important to understand how market, socio-economic, and institutional factors interact with each other to enhance or constrain market participation and crop productivity of smallholder farmers, which in turn affect household dietary diversity. The conceptual framework provided in this study shows that smallholder farmers are affected by many factors, which can be grouped into three categories (market, socio-economic, and institutional factors). It was conceptualized that socio-economic and institutional factors impact market participation and crop productivity, while market factors mainly affect market participation. These factors’ positive influence on smallholder farmers leads to improved crop productivity (more crops produced), which then leads to high market participation. High market participation results in increased income, leading to improved food security, dietary diversity, and sustainable livelihoods. Figure 1 illustrates the interrelationship among the key variables in the study.

### 2.4 Measuring Dietary Diversity (HDDS)

The Household Dietary Diversity Score (HDDS) was employed in this study, including 12 food groups [34]. HDDS was derived using data from the 24 h food consumption recall. Since households often diversify their food consumption patterns with increasing earnings and when they have attained specific minimum levels of calorie sufficiency, HDDS is a useful proxy for a household’s economic access to food and food security [10]. The HDDS represents the range of food and dietary diversity available to a household [35]. The standard 12 food categories (cereals; vegetables; roots and tubers; fruits; fish and seafood; pulses/legumes/nuts; milk and milk products; oil/fats; sugar/honey; eggs; legumes, nuts, and seeds; and spices, condiments, and beverages) specified by Swindale and Bilinsky [34] were followed. Food consumed outside the household, such as at restaurants, social gatherings, or any other unique occurrences, is not included in this category because it would otherwise misrepresent routine and typical food intake at the household level. The HDDS in this study was used rs an outcome/dependent variable for both the subsistenœ (crop productivity) and market (market participation) pathways.

### 2.5 Measuring the Effect of Crop Productivity on Household Dietary Diversity

To determine the influence of crop productivity on household dietary diversity, the following equation was used, where a smallholder farmer is a unit of analysis [36–38]:

(1) $$y_{i}=\beta_{0}+\beta_{1}\times CP_{i}+\beta_{2}X_{i}+\eta_{i}+v_{i}$$

where *y_i_* is a measure of household dietary diversity *i*; *CP_i_* is a binary variable taking 1 if smallholder farmer *i* had crop productivity and 0 otherwise; *X_i_* is a vector of household characteristics; *η_i_* is a term capturing unobserved heterogeneity assumed to be unrelated to the explanatory variables vector *X_i_* and refers to smallholder farmers who are from the same area; and takes all the outstanding variation with *v_i_* ~ IIDN(0,1).

If the vector *X_i_* contains all of the predicted crop productivity components, including location fixed effects, and is uncorrelated with the error *V_i_*, then an ordinary least squares (OLS) regression of Equation (1) will produce consistent results. In such situation, the coefficient of interest *β*_1_, which quantifies the influence of crop productivity extent, can be viewed as the genuine impact of crop productivity on the dietary diversity of smallholder farmers.

The crop productivity of smallholder farmers is affected by many unobserved factors, making it an endogenous variable, and failing to account for this endogeneity may result in biased and inconsistent estimates. Crop production endogeneity happens when some smallholder farmers have the skills and resources to boost crop productivity while others do not. In a regression model of the type provided in Equation (1), this type of selection bias will exaggerate the actual influence on crop productivity. On the other hand, unprivileged smallholder farmers may be unable to boost crop output due to a lack of agricultural inputs. In this case, failing to account for this type of bias understates the ostensible genuine benefit of crop productivity. Crop productivity (*CP*), a potentially endogenous variable, takes the following form [36]:

(2) $$CP_{i}^{*}=\alpha_{0}+\alpha_{1}Z_{i}+\alpha_{2}X_{i}+\eta_{2}+\varepsilon_{i}$$

where $CP_{i}^{*}$ is the propensity to increase crop productivity. However, $CP_{i}^{*}$ is unobserved and what we observe instead is the following:

(3) $$\begin{array}{ll} CP= & \{\begin{array}{ll} 1 & \;if\;Cropproductivityscore\;>0\\ 0 & \;otherwise\; \end{array} \end{array}$$

The vector *Z_i_* includes a number of variables that influence crop productivity, such as smallholder farmers’ management and technical ability, as well as government agricultural support [39,40]. *η*_2_ is the unobserved heterogeneity parameter, which is believed to be uncorrelated with the vector of explanatory factors (*X_i_*) and *ε_i_* represents the remaining unobserved variance. The unobserved heterogeneity components (*η*) contains subscripts {1, 2} which are equation indicators.

Following previous studies [36–38,41], the Conditional Mixed Process (CMP) was used to account for the possibility of endogeneity bias. Allowing for crop productivity endogeneity in Equation (1), the joint marginal likelihood can be observed as follows [37,38]:

(4) $$\int_{\eta_{2}}\int_{\eta_{1}}\left[\prod^{\text{​}}L_{2}(\eta_{2})\prod^{\text{​}}L_{1}(\eta_{1})\right]f(\eta_{2},\eta_{1})d\eta_{2}\eta_{1}$$

where *L*_1_ and *L*_2_ are the conditional likelihood functions of Equations (1) and (2) respectively; *f*(*η*_2_, *η*_1_) is the joint distribution of the unobserved heterogeneity components. In this case, the joint distribution of the unobserved effects *f* (*η*_2_, *η*_1_) is assumed to be a two-dimensional normal distribution characterized as follows:

(5) $$\left(\begin{array}{l} \eta_{2}\\ \eta_{1} \end{array}\right)∼N\left(\left[\begin{array}{l} 0\\ 0 \end{array}\right],\left[\begin{array}{l} \sigma_{2}^{2}\\ \rho_{12}\sigma_{2}\sigma_{1}\sigma_{1}^{2} \end{array}\right]\right)$$

The conditional mixed process (CMP), which uses the Geweke, Hajivassiliou, and Keane (GHK) algorithm to consistently estimate the likelihood function specified in a model [42], was used to jointly estimate the complete specification or full model (Equation (3)). The fundamental purpose of evaluating Equations (1) and (2) jointly was to account for any selfselection bias. According to Maitra [42], joint estimation shows the possibility of non-zero covariance between the error terms of Equations (1) and (2), i.e., *cov*(*η*_2_, *η*_1_) ≠ 0. However, because we stated the heterogeneity factors, Equations (1) and (2) become independent, making the likelihood function in Equation (3) above easily obtained by simply multiplying the individual conditional likelihood functions of Equations (1) and (2) [43].

### 2.6 Measuring the Effect of Market Access on Household Dietary Diversity

The study also assessed the impact of market participation on household dietary diversity. Smallholder farmers that participated in the market had the capacity to earn money and buy more nutrient-dense food to satisfy their daily dietary needs. Market participation could be determined by looking at farmers’ incomes and sales. Smallholder farmers that participated in the market were deemed market participants and received a score of 1, otherwise, they received a score of 0.

The study used the instrumental variable Poisson regression model to address the confounding factors and the issue of counterfactuals. The model accounted for both observed and undiscovered heterogeneity [44,45]. The count outcome with a Poisson error-term distribution was used in the model to assess the causal effect of market participation on nutrition security status. This study aims at estimating the average treatment effect on the treated (ATT). According to Takahashi and Barrett [46], the ATT is the average difference between smallholder farmers’ potential results with and without access to the market. Imbens and Wooldridge [47] and Adolwa et al. [48] suggested that the ATT can be illustrated as follows:

(6) $$ATT=E(Y_{1j}-Y_{0j}/T_{j}=1)=E(Y_{1j}/T_{j}=1)-E(Y_{0j}/T_{j}=1)$$

where *E*{.} denotes the expectation operator, *Y*_1*j*_ is the potential outcome for smallholder farmers who participate in the market, *Y*_0*j*_ is the potential outcome of smallholder farmers who do not participate in the market. *T_j_* represents the treatment indicator which takes the value 1 if smallholder farmers participate in the market and 0 otherwise. Unobserved counterfactual events were a major impediment to predicting the ATT. As a result, it was nearly impossible to predict the potential outcomes of farmers who participated in the market if they had not participated. Replacing this unobserved counterfactual with the possible outcomes of smallholder farmers who have not participated in the market is also impractical because it is likely to result in biased estimates [46]. The problem is addressed using the primary model by Terza [49], the endogenous Poisson treatment effect.

#### Endogenous Treatment Effect Model for a Count Outcome—Poisson

As noted previously, the study’s goal is to determine whether smallholder farmers’ market participation affects their dietary diversity. Because smallholder farmers’ market participation is not exogenous, it is considered an endogenous binary-treatment variable *T_j_*. *T_j_* Is endogenous if the treatment assignment is not random, but some unobservable covariates (variables) affect *T_j_* which also impact the outcome variable. Since the HDDS (outcome variable) is a count event that takes values, *Y_j_* = 0,1,2,… *Y_n_*, and smallholder farmers choose whether to adopt one or none, a second dummy *S_j_* was developed to represent a sample selection rule. That is, smallholder farmers may not be able to engage in the market. In this case, *S_j_* is missing for a proportion of the sample and the selection rule is defined such that *S_j_* = 1 when *Y_j_* is observed and *S_j_* = 0 when *Y_j_* is missing. The matter of endogeneity and sample selection was solved using the count data model with endogenous treatment [19].

The Poisson endogenous treatment effect model considers the case in which the selection dummy is assigned the value 0 when smallholder farmers did not receive nutritional security status from market participation (*Y_j_* is missing) and 1 when smallholder farmers did receive dietary diversity from market participation (*Y_j_* is observed). Continuous latent variables can be used to generate selection dummies and endogenous treatments, as shown below [19]:

(7) $$T_{j}^{*}=Z_{i}^{\prime }+\mu_{j}$$

(8) $$S_{j}^{*}=X_{j}^{\prime }\beta +\delta T_{j}+\varepsilon_{j}$$

With $T_{j}=1(T_{j}^{*}>0)$, $S_{j}=1(S_{j}^{*}>0)$, the outcome model, which follows a Poisson distribution, can be specified as:

(9) $$Y_{j}=\left\{0\left\{\mu^{Y_{j}}\exp(-\mu )\right\}/Y_{j}!\begin{array}{l} \;if\;S=0\\ \;if\;S=1 \end{array}\right\}$$

Thus, $E\left(\frac{Y_{j}}{X_{j}},T_{j},\varepsilon_{j}\right)=\exp(X_{j}\beta +\delta T_{j}+\varepsilon_{j})$.

*X_j_* indicates the covariate vector used to model the count outcome; *Z_j_* is the covariates for binary treatment; *ε_j_* and *μ_j_* are the error terms for the outcome and treatment, respectively. The two error terms have a mean of zero and are bivariate normal. Since the covariates *X_j_* and *Z_j_* are exogenous, they are unrelated to the error terms. Conditional on *ε_j_*, *μ_j_* is normal with mean *ε_jρ/σ_* and variance (1 – *ρ*^2^). The endogenous treatment Poisson regression model is nested in a possible outcome model to estimate the ATE and ATT. The prospective outcome model describes what each farm household might receive at each treatment level.

##### 1 Description of other key variables

Dietary diversity in smallholder farm households is not only influenced by crop productivity and market participation but also by a number of other farm, household, and contextual characteristics. Some of these characteristics may be linked to crop productivity or market participation, so we need to control for them in the regression models to avoid estimation bias. Age of household head, gender of household head, marital status, educational level of household head, household size, disability in the family, income, ownership of livestock, and irrigation system were identified as socioeconomic and internal factors. These factors mainly affect the production, management, and harvesting systems of farming and the decision of smallholders to be involved in crop production [50]. Access to market information, access to agricultural assistance, and social grants were identified as external variables [50]. These factors affect smallholder farmers’ ability to produce more and to participate in the market. These variables were shown to influence household diets and nutrition in previous studies [19,51].

## 3 Results and Discussion

### 3.1 Descriptive Analysis of the Results

Summary statistics of the socio-demographic factors used in this study are provided in this section, and the percentages consuming the different food groups are shown in Table 1. The results showed that 73% of participants were male-headed while 27% were female-headed. In terms of educational level, 47% of the participants had a secondary education, followed by 27% who had no schooling, while only 4% had gone to tertiary. About 31% of the smallholder farmers were single and had never been married, followed by 23% who were legally married, and only 2% were separated but still legally married. The results also revealed that 21% of smallholders had a disability in their families, while 79% did not. In terms of HIV status, only 9% of smallholder farmers had a family member with HIV, while 91% did not. About 37% of smallholder farmers received a social grant, while 63% did not receive any social grants.

The results showed that out of the total sample size, only 25% of the smallholder farmers were involved in crop production, while 75% were not. Regarding market participation, about 14% of the smallholder farmers participated in the market, while 86% did not participate. About 23% of smallholder farmers owned livestock, while 77% did not own any livestock. In terms of agricultural assistance, only 6% of the smallholder farmers received assistance, while 94% did not. The result also showed that 92% of smallholder farmers did not have access to market information, while 8% did. About 48% of the smallholder farmers had access to the irrigation system, while 52% did not. The results also revealed that the average household size was five. Furthermore, the average age of the participants was 49 years old.

### 3.2 Different Food Groups Consumed by Smallholders in Mpumalanga and Limpopo Provinces

Dietary diversity scores are based on the number of specified food groups consumed over a given period (as shown in Table 1). Cereals were the most-consumed food group (98%) in the 7-day recall period, followed by spices, condiments, beverages (95%), and oil/fats (93%), as shown in Table 1. The results showed that sugar/honey was consumed by 77% of the smallholder farmers. Approximately 73% of the smallholder farmers consumed Pulses/legumes/nuts. Legumes, nuts, and seeds were consumed by 62% of smallholder farmers. The results showed that vegetables and fruits were the least consumed food groups, at 37% and 23%, respectively.

### 3.3 Dietary Diversity of Smallholder Farmers

Table 2 shows the dietary diversity of smallholder farmers in the Limpopo and Mpumalanga provinces before the study was conducted. Using the cut-offs recommended by Kennedy et al. (2011), the overall sampled population (*n* = 1520) showed that 57% of smallholder farmers consumed highly diverse diets (more or equal to 6 food groups), whereas 25% and 18% of smallholder farmers consumed medium dietary diversity (4–5 food groups) and low diverse diets (less or equal to 3 food groups), respectively. Although more than half (57%) of smallholder farmers consumed highly diverse diets, cereals were the food most consumed. This means that even though smallholder farmers diversified their consumption, cereals dominated their diet. This is because cereals are staple crops in many rural households and are grown the most. The main staple food of Kenya was maize, which accounted for about 65% of the total staple food calorie intake and 36% of the total food calorie intake [52]. Beyer [53] reported that an unbalanced diet arises from a dependency on micronutrient-deficient staple foods, and the predominant consumption of staple crops without diversification or the use of supplements results in severe poverty.

### 3.4 Impact of Crop Productivity on the HHDS (Dietary Diversity) of Smallholder Farmers—Conditional Mixed Process (CMP) Model

Table 3 displays the estimation results obtained by combining Equations (1) and (2). As noted previously, joint estimation of the system of equations allows us to account for endogeneity bias in the equations of dietary diversity caused by crop productivity selectivity bias (HDDS). The key measure of selection bias is the atanhrho reported at the bottom of Table 3. To make the rhos (*p*) unbounded, the stated atanhrho value is the arc-hyperbolic tangent of the rhos (*p*). A positive atanhrho value indicates that some unobservable factors are influencing crop productivity and the main outcome variable. In this study, the atanhrho_ 12 was significant and negative in the outcome equations. The negative atanhrho_ 12 result indicates that no omitted variables influenced either outcome variable.

The CMP model results in Table 3 showed that household size had a positive and significant impact on dietary diversity. The positive relationship between household size and dietary diversity suggests that the majority of households were old enough to participate in production and marketing. The work on farms and in the market was shared among smallholder farmers, which saved time and money [50]. However, according to Olayemi [54], a large family size means more children in the family, which affects the food intake and dependency ratio. The study concluded that the larger the household size, the less food is available for each family member, and nutritional status suffers. Tsegay [55] agreed with Olayemi [54], reporting that increasing family size puts more pressure on food consumption than the benefit of additional labor for production. Furthermore, Muche et al. [56] found that larger family sizes put additional strain on household food security by increasing food and non-food expenditures.

Social grants are becoming increasingly popular to improve the well-being of poverty-stricken households in South Africa and elsewhere [57]. However, the findings show an adverse relationship between dietary diversity and social grant on crop productivity. One of the plausible causes is that some smallholder farmers who receive social grants do not want to participate in crop production and do not spend their funds on healthy food. In response to this finding, Boone, Covarrubias [58], and Sinyolo et al. [59] explained that social grants discourage many households from participating in agricultural production. The majority of households regard social grants as their primary source of income and disregard farming. This has resulted in less dietary diversity in their food, as social grants are insufficient to help them afford nutritious food. Social grant income ranges between R350 and R1980 in South Africa. This is not enough to meet all the needs of the households.

In this study, “output” referred to the yield obtained by smallholder farmers during crop production. The quantity of yield determines whether farmers can consume and sell. The study discovered unexpected results about the impact of output. Smallholder farmers’ overall output had a negative and significant impact on their dietary diversity. This means that smallholder farmers faced numerous challenges that prevented them from producing enough diversified crops. They end up producing less diversified crops, mainly for their own consumption. Therefore, they were not making any income to buy other food groups required for a balanced diet. On the contrary, Liliane et al. [60] discovered that increased crop yields reduced poverty and malnutrition significantly. According to the study, crop yield is influenced by various factors in a specific area. Agricultural practices, managerial decisions, diseases and pests, climatic conditions, soil fertility, and topography are among the factors. As a result, farmers must understand all these factors before beginning crop production.

### 3.5 The Impact of Determinants of Market Participation on the Dietary Diversity of Smallholder Farmers—Poisson Endogenous Treatment Effect Model

According to the Chi^2^ value (92.77, *p* < 0.001), the model is statistically significant at 1%, indicating a good fit. At 1%, the rho (*ρ*) was statistically significant (0.998, *p* < 0.002). The significance of the rho (*ρ*) implies that smallholder farmers’ dietary diversity is affected by unobserved characteristics that influence their market participation decisions. To address the issue of endogeneity, the Poisson endogenous treatment effect model was used. As shown in Table 4, livestock ownership, social grant, wealth index, access to market information, and crop production involvement were all statistically significant.

Livestock is a significant production shifter because it increases a household’s ability to produce more, increasing the probability of market participation [61]. According to this study’s findings, smallholder farmers’ dietary diversity was positively and statistically significant when they owned livestock. Smallholder farmers with livestock can sell some of their livestock to buy nutritious foods while increasing crop cultivation. However, Kyaw et al. [62] observed that if a household owns livestock, the household members must split time and money with the livestock for feeding and caring for the livestock, resulting in less production surplus to sell in the market. According to the authors, farmers with inadequate land must forgo crop output to concentrate on livestock production, which may negatively impact their marketable excess.

The findings revealed that social grants had a positive and statistically significant (p > 0.01) impact on smallholder farmers’ HDDS on the market participation side. This means that some smallholder farmers could use their social grants effectively to participate in the market. Several studies reported that social grants could increase rural households’ economic strength; however, many rural households misuse social grant funds [58,59,63].

Smallholders’ wealth index had a positive and significant impact. The findings also revealed that smallholder farmers’ involvement in crop production was statistically significant at 1% and positively impacted their HDDS. This is because smallholder farmers who produce crops take advantage of market participation by acting as sellers and buyers. They grow a wider range of crops to sell the surplus in the market and use the proceeds to purchase other food groups they cannot produce. More crop production increased the ability of smallholder farmers to use the inputs they have to produce more output at a given time. This finding is consistent with the findings of Mathenge et al. [64] and Mulenga et al. [65], who found that farmers involved in crop production have more comparative advantages in resource use, which can be seen in improved productivity through economies of scale.

The coefficient on access to market information positively affected the HDDS and was significant at the 5% level. Market information’s positive outcome suggests that farmers with access to market information are more likely to sell their products and make a profit. The findings revealed that market information greatly assisted farmers’ market knowledge. Farmers were able to obtain information on pricing strategies as well as information on highly regarded crops. This finding is consistent with Kyaw et al. [62], who deduced that access to market information leads to increased productivity and a high marketable surplus.

#### Treatment Effects on Market Participation of Smallholder Farmers

This research aimed to see how market participation affected smallholder farmers’ dietary diversity in terms of HDDS. The results showed that the average per capita HDDS of smallholder farmers who participated in the market was higher (2.134) than that of farmers who did not participate in the market (1.982). A simple significant difference in average per capita HDDS between market participants and non-market participants is misleading because it involves bias and fails to account for potential heterogeneity in the two groups’ characteristics. Even though it accounts for endogeneity, the evaluation from the endogenous Poisson regression model may be insufficient. Because the issue of missing data (counterfactual scenario) has not been considered, direct coefficients from the model cannot be considered as average treatment effect on the treated (ATT).

As a result, the focus of this study was on the effects of market participation on smallholder farmers’ dietary diversity in terms of HDDS using ATT and ATE, with Poisson regression and endogenous treatment effects employed. After fitting the Poisson regression with endogenous treatment effects, the ATE and ATT were calculated. The estimated potential outcome means (ATE) of market participation on HDDS were approximately 0.747, as shown in Table 5 and were statistically significant at 1%. According to the ATE estimate, the average smallholder farmer who participated in the market among the entire sampled population had improved dietary diversity. Similarly, the conditional treatment effect on HDDS, which measures the ATT of market participation, was around 0.768 and statistically significant at 1%. As a result, smallholder farmers who participated in the market had approximately 0.768 more HDDS than farmers who did not participate in the market.

## 4 Conclusions and Policy Recommendations

Crop productivity and market participation have the potential to improve dietary diversity and nutrition quality among smallholder farmers effectively; however, numerous challenges threaten the prospect of smallholder farming. The study assessed the role of subsistence farming in crop productivity and market access on rural household dietary diversity. The findings showed that household size, ownership of livestock, wealth index, and involvement in crop production positively influenced household dietary diversity. On the other hand, the output showed a negative effect. The results also showed that social grants had contradicting results; they had a negative impact on the HDDS received from crop productivity while having a positive effect on the HDDS from market participation. It can be concluded that smallholder farmers who receive social grants were reluctant to use the money as inputs to production; however, they used it to enhance their participation in the market. Providing ways in which smallholder farmers can use their funds effectively can help to improve household dietary diversity and nutrition quality.

The study recommends that smallholder farmers should be given more training on allocating the funds and resources to increase their standard of living. This can be achieved by conducting more workshops and training covering all the sustainable production systems that smallholder farmers can undertake to produce different types of food groups. These will raise awareness among smallholder farmers about the required balanced diets for food and nutrition security. Smallholder farmers should also be given lessons and training that will teach them how to use their funds and resources to help them generate income. Even though the recommendations were implemented before, smallholder farmers still face a high malnutrition rate. This shows a need to adjust and monitor their implementation to ensure that they serve the intended purpose.

Furthermore, to facilitate informed decision-making regarding farming and marketing landscapes, smallholder farmers should be encouraged to keep up with media communication platforms. Therefore, it is important to empower the smallholder farmers to be literate to use media platforms. Smallholder farmers should obtain updated information on all the digital devices (TV, radio, cellphones, laptops, tablets, etc.) available to them so as to improve their decision-making. Extension officers can also update smallholder farmers through the use of digital platforms. Smallholder farmers need to have frequently updated information on the type of crop demand in the market and the pricing strategies that they can use to make a sufficient income.

## 5 Limitations of the Study and Directions for Future Research

The data used in this was collected during the 2016/2017 season. Since then, smallholder farmers’ production and marketing systems may have changed. Smallholder agriculture is dynamic and affected by many factors, so it is important to frequently conduct research and use the most recent findings. Future studies can use primary and secondary data to compare the findings before and after the spread of COVID-19 to see how the nutrition security status of rural households was affected. The spread of the corona virus has pushed many smallholder farmers into food and nutritional insecurity. The government’s COVID-19 regulations, which included restrictions on movements, the closure of borders, physical social distancing, the closure of informal markets, and the closure of various livelihood opportunities, pushed households to be more food insecure. While the placement of these regulations was mainly for health measures, they also indirectly affected the economic and agricultural sectors. To reduce the impact of the pandemic on poverty and food insecurity, the government in South Africa provided financial and food assistance as measures to improve livelihoods. There is a need to use longitudinal data to show the constant changes in factors that affect smallholders, such as malnutrition. This study focused on two provinces in South Africa. There is a need to broaden the research to include all provinces and compare crop productivity and market participation across provinces to draw lessons from all of them. The findings can be used to compare smallholder farmers’ challenges and opportunities in their different working environments. The findings will also be used to identify the areas for improvement that each province needs.

## Figures and Tables

**Table 1:** Food groups consumed by households in Mpumalanga and Limpopo provinces in the past seven days.

Food Groups	Frequency	Percentage (%)
Cereals	1490	98
Vegetables	562	37
Roots and tubers	790	52
Fruits	350	23
Fish and seafood	851	56
Pulses/legumes/nuts	1110	73
Milk and milk products	866	57
Oil/fats	1414	93
Sugar/honey	1170	77
Eggs	882	58
Legumes, nuts, and seeds	942	62
Spices, condiments, beverages	1444	95

**Table 2:** Dietary diversity of smallholder farmers in Limpopo (n = 911) and Mpumalanga (n = 609) Provinces, South Africa.

Dietary Diversity Categories	Percentage (%)
Lowest dietary diversity	18
Medium dietary diversity	25
High dietary diversity	57

**Table 3:** Determinants of crop productivity on dietary diversity using Conditional Mixed Process (CMP) model.

	HHDS (Dietary Diversity)		
Variables	Coef.	Std.Err.	*p* Value
Age	–0.002	0.002	0.412
Household size	0.052	0.016	0.001 ***
Social grant	–0.254	0.144	0.077 *
Agricultural assistance	–0.647	0.167	0.544
Ownership of livestock	0.187	0.476	0.694
Educational level of household head	0.176	0.590	0.765
Government advice	–0.203	0.175	0.246
Harvest (kg)	–0.030	0.016	0.051 **
Disability in the family	0.300	0.467	0.520
Economic activity	0.355	0.219	0.106
Gender	0.849	0.682	0.213
Family member with HIV	0.029	0.407	0.943
Cut_1	–0.859	0.167	0.000
Cut_2	1.663	0.172	0.000
Atanhrho_12	–0.081	0.048	0.090
rho_12	–0.080	–0.172	0.013

Notes: ***, **, * Indicate statistical significance at 1%, 5%, and 10% level, respectively.

**Table 4:** Determinants of dietary diversity using Poisson regression with endogenous treatment.

Variables	Coef.	Std.Err.	p Value
HDDS			
Age of the household head	–0.000	0.001	0.583
Gender of household head	–0.009	0.022	0.676
Households size	0.009	0.002	0.656
Educational level of household head	–0.047	0.054	0.391
Marital status	0.023	0.054	0.667
Access to agricultural assistance	–0.090	0.014	0.546
Ownership of livestock	0.123	0.057	0.030 **
Family member worked for a wage salary	0.009	0.043	0.838
Social grant	0.038	0.020	0.056 *
Wealth index	0.058	0.024	0.015 **
Access to market information	0.038	0.018	0.031 **
Involvement in crop production	0.199	0.058	0.001 ***
Family member with HIV	0.004	0.052	0.687
Market participation	0.084	0.029	–2.950
_constant	2.066	0.095	21.730
Market participation			
If household received agricultural-related assistance	2.592	0.028	93.210
_constant	–0.931	0.015	–62.570
/athrho	3.430	0.374	9.170
/lnsigma	–17.326	0.107	–161.710
Wald Chi^2^ (15)	92.77	0.000	
rho (*ρ*)	0.998		
	0.002		
sigma (*σ*)	0.000		
	0.000		

Notes: Dependent variable is HDDS; ***, **, * indicate significance at 1%, 5%, and 10% levels, respectively. Source: Authors’ own analysis.

**Table 5:** Treatment effects on market participation of smallholder farmers.

Treatment Effects	Coefficient	Std.Err.	*p* Value
Poisson regression with treatment effects			
Average treatment (ATE)	0.747	0.267	0.003 ***
Average treatment effect on the treated (ATT)	0.768	0.255	0.004 ***

Notes: *** indicate significance at 1%level, Source: Authors’ own analysis.

## Data Availability

Restrictions apply to the availability of this data. Data were obtained from the Department of Agriculture, Land Reform, and Rural Development (DALRRD) and are available from the South African Vulnerability Assessment Committee (SAVAC) secretariat with the permission of the Department of Agriculture, Land Reform, and Rural Development (DALRRD).
